# From Pandemic to Endemic–Redefining Excellence in Thoracic Surgery Service Delivery

**DOI:** 10.3389/fsurg.2021.741366

**Published:** 2021-12-10

**Authors:** Lowell Leow, John Kit Chung Tam

**Affiliations:** ^1^Department of Cardiac, Thoracic and Vascular Surgery, National University Heart Centre Singapore, Singapore, Singapore; ^2^Department of Surgery, Yong Loo Lin School of Medicine, National University of Singapore, Singapore, Singapore; ^3^Lung Surgery Centre, National University Health System, Singapore, Singapore

**Keywords:** COVID-19, thoracic and cardiovascular surgery, lung surgery, pandemic (COVID-19), endemic

## Abstract

Covid-19 has touched all corners of the globe and impacted our lives in more ways than one. Thoracic surgeons are frontliners impacted in both our professional and personal capacities. In this commentary we discuss the impact that Covid-19 has had on thoracic surgery as a practice highlighting the discrepant impact upon developed and developing countries, the state of affairs of the “new normal” that we live in and the challenges ahead as we transition from pandemic living to endemic living alongside Covid-19. We need to evolve as the virus does and keep abreast of the latest developments to continue providing excellent care to our patients. While the challenges brought about by the Covid-19 pandemic are unprecedented in this generation, it can bring forth tremendous opportunities for us to redefine excellence in thoracic surgery service delivery in this endemic times.

## Introduction

It has been 21 months since the Covid-19 first entered our communities, causing widespread changes to our lives, livelihoods, and clinical practices. Despite ongoing global vaccination efforts and universal adoption of social distancing measures, the virus is mutating, and new strains constantly keep us on our toes. Our battle with the Covid-19 pandemic will eventually transition into an endemic one (1, 2). Fortunately, even as the virus mutates and vaccinations get rolled out, its potency appears to wane. We will be left to grapple with its continued presence and the post pandemic consequences in the years to come (3).

## Left Behind

The Covid-19 pandemic has laid bare and accelerated the geopolitical and economic divide between developed and developing countries. From the ability to muster an initial response, to testing capabilities and now vaccine acquisition and adoption, there is a stark difference between resource-rich and poorer countries. While there appears to be a concentration of cases and corresponding deaths in the developed nations, one cannot help to wonder the possibility of under-detection and under-reporting in developing countries, and by extrapolation the real impact Covid-19 is having on these countries and their health systems (4). Essential medical equipment, beds in hospital wards and intensive care units, oxygen supply, ventilator machines, and healthcare workers are all in short supply. Elective medical care has taken a backseat during the pandemic and there is a significant backlog of thoracic surgical cases as each country's health system struggles against global healthcare resource shortages. This has resulted in a tremendous care deficit in many countries with large number of patients unable to receive the surgeries they needed.

While there have been guidelines published on the prioritization of lung cancer patients during the pandemic (5, 6), it is conceivable that some patients are neglected and fallen behind in countries where resources are over expended. Also, lockdowns may have prevented patients from undergoing necessary investigations for earlier diagnosis. As a result of a delay in care, there are plausible risks that their thoracic malignancies may have progressed. Even if patients had received neoadjuvant therapy to defer surgical intervention by up to 3 months (5), proper restaging work up and discussion at a multi-disciplinary forum should be performed. Some patients may inevitably experience disease progression and become non-surgical candidates during this period, hence surgeons may need to triage and prioritize patients according to the severity of their condition, taking care not to delay further those who have completely resectable diseases. Provision of elective thoracic surgical oncology service should be ramped up as soon as possible when Covid-19 is better under control to catch up and clear the backlog present.

## The New Normal

Much has been written about organizing thoracic surgical services during a pandemic (6–8), but increasingly it appears inevitable that we will need to resume our duties and services in the presence of Covid-19. This is not without its risks: patients with Covid-19 infections are at higher risk of postoperative pulmonary complications which are associated with higher mortality (9). Thoracic patients who undergo lung resections have reduced pulmonary reserves and are naturally at higher risks for morbidity and mortality should they suffer a respiratory insult. This calls for heightened vigilance for our patients to ensure the upholding of our pre-pandemic standard of care. Careful screening of patients for signs and symptoms of coronavirus infection should become routine and a new standard of care. All inpatients should be tested for Covid-19 upon admission to the hospital. Regular Covid-19 testing for healthcare staff should be considered to prevent the spreading of coronavirus within a hospital cluster. All healthcare workers should be protected against Covid-19 by full vaccination. Clinical management should be tailored toward outpatient setting where possible to minimize exposure risk to patients and preserve hospital resources that may be stretched (10). Enhanced Recovery After Surgery (ERAS) protocols and minimally invasive thoracic surgery techniques should be widely adopted into local thoracic surgical practices to facilitate fast recovery and early discharge from hospitalization (11).

The wellness and safety of healthcare staff is paramount and must be protected (12, 13). As thoracic surgery is an aerosol-generating procedure (14), we need to be mindful to ensure appropriate personal protective measures are taken during and after surgery to avoid exposure to our staff (7, 12, 15). In the event of personal protective equipment (PPE) shortages, allocation and utilization of PPE may have to be rationalized. Contact and droplet precautions must always be observed (13). The physical exhaustion, psychological impact, and emotional trauma experienced by caregivers during the Covid-19 pandemic are real. Beyond providing for the physical needs of our staff, leaders must also support the psychological and emotional well-being of their team members and offer suitable avenues for respite and restoration (12, 16).

This pandemic has catalyzed the digitization of healthcare and we should continue to embrace it. Virtual based care, teleconsultations, home-based nursing and rehabilitation therapy, and home delivery services for prescription medicines can help to reduce footfall and congregation in the hospitals where Covid-19 patients are more likely to be encountered (3, 7, 10). From automated patient servicing counters to electronic hospital apps to virtual staff meetings, even the least tech-savvy of us will have to eventually accept this new normal (3, 7).

## Challenges Ahead

As countries rapidly push to vaccinate the population en masse (4), patients will increasingly look to us for advice regarding vaccine efficacy and safety. Unvaccinated individuals are at multi-fold higher probability of contracting COVID-19, and vaccination is now considered as a new public health standard that is often strongly encouraged or even mandated in most jurisdiction. By virtue of limited lung reserves, all our patients are at higher risk for severe respiratory sequelae should they contract Covid-19. As thoracic specialists, we need to keep abreast of the evolving vaccination trends and the latest literature to offer our patients the best medical advice. For patients who have potential contraindications against taking the vaccine, thoracic surgeons should balance the risks vs. benefits and advise them according to their individualized medical conditions. Based on the known safety profile of the vaccines, current recommendations allow as short as 2 weeks before and after surgery for vaccine administration (17). Patients with solid cancers undergoing chemotherapy or radiotherapy are also now recommended to get the vaccine (17). The long-term efficacy of vaccination is yet uncertain. Some reports show waning antibody-mediated immunity after 6 months, and booster vaccine shots is emerging to become an important measure in some countries (18).

The pandemic has facilitated this drive for updated knowledge dissemination. There are a plethora of international webinars and telecasts that have made information easily accessible (3, 7). From a time when keeping up to date was a costly and time-consuming affair, we now have an abundance of virtual educational programs available at our fingertips. At times like this, we need to remain rooted in our evidence-based fundamentals and be discerning of the information encountered, so as to translate it to best care for patients in our local setting. As more specialists adopt a larger online presence, networking becomes more convenient and collaborations are less cumbersome than before (3). In the face of Covid-19, we need to continue collaborating and innovating to advance the frontiers in thoracic surgery.

Covid-19 will be known as the defining event in modern history. Given how widely Covid-19 has spread, it is unlikely that the virus can be completely eradicated, and we will have to learn to live with it in our midst. Covid-19 is changing from a pandemic to endemic disease within our environments and communities. Thoracic surgeons must fully embrace innovation, digitalization, automation, and vaccination in this post-Covid-19 reality. From streamlining case selection, to optimizing infection control, to minimizing surgery invasiveness and enhancing recovery pathways, excellence in thoracic surgery service delivery in this new era will be redefined by successful adaptation, relentless innovation, and “doing all things well” to deliver the best possible care and outcomes to our patients (Table 1). COVID-19 tests our ingenuity as thoracic surgeons to come up with new and improved ways to serve our patients. With the proud heritage of innovation and adaptability in our specialty, we are certain that we will rise to this challenge of our generation.

**Table 1:** Summarizing prominent issues and proposed solutions.

**Issues**	**Solutions**
SARS-CoV-2 virus mutates constantly	Keep abreast of latest developments of Covid-19 to better address patient specific needs
Discrepant response in resource poor countries	Outreach programmes, vaccine sharing programmes extended by resource rich countries
Delay in surgical treatment due to Covid-19 limitations	Ensure proper restaging and discussion at multi-disciplinary forum before surgery to avoid potential harm
Transitioning healthcare system from pandemic to endemic stance	Guideline and multi-disciplinary driven decision for prioritization of patients for surgery based on tumor biology
Working alongside Covid-19	Continued vigilance and compliance to personal protective equipment and surveillance swabs
Burnout from prolonged heightened alert	Scheduled breaks and regular checks on mental wellness of staff. Foster kindness and camaraderie in supporting each other.
Maintaining continuity of care whilst observing social distancing practices	Embrace the adaptation of digital technology to facilitate patient encounters and staff meetings
Suspension of scientific meetings due to travel restrictions	Webinars and telecasts that can now reach a wider audience
Limited hospital resources and reduced elective surgery beds	Accelerated adoption of minimally invasive techniques and enhanced recovery protocols to minimize surgical morbidity and increasing patient throughput

## Data Availability Statement

The original contributions presented in the study are included in the article/supplementary material, further inquiries can be directed to the corresponding authors.

## Author Contributions

JT was invited for submission, conceived the paper, revised, and edited the final draft. LL formulated the first draft. All authors contributed to the article and approved the submitted version.

## Conflict of Interest

The authors declare that the research was conducted in the absence of any commercial or financial relationships that could be construed as a potential conflict of interest.

## Publisher's Note

All claims expressed in this article are solely those of the authors and do not necessarily represent those of their affiliated organizations, or those of the publisher, the editors and the reviewers. Any product that may be evaluated in this article, or claim that may be made by its manufacturer, is not guaranteed or endorsed by the publisher.
